# Prognostic Significance of Isolated Low-Frequency Hearing Loss: A Longitudinal Audiometric Study

**DOI:** 10.3390/jcm14196749

**Published:** 2025-09-24

**Authors:** Junhun Lee, Chul Young Yoon, Jiwon Kim, Young Joon Seo

**Affiliations:** 1 Research Institute of Hearing Enhancement, Yonsei University Wonju College of Medicine, Wonju 26426, Republic of Korea; junhun0702@naver.com (J.L.); fezro@yonsei.ac.kr (C.Y.Y.); flora4820@yonsei.ac.kr (J.K.); 2 Department of Medical Informatics and Biostatistics, Yonsei University Wonju College of Medicine, Wonju 26426, Republic of Korea; 3 Department of Otorhinolaryngology, Yonsei University Wonju College of Medicine, Wonju 26426, Republic of Korea

**Keywords:** hearing loss, pure-tone audiometry, low-frequency hearing loss, high-frequency hearing loss, combined-frequency hearing loss, long-term outcomes, risk stratification, logistic regression

## Abstract

**Background/Objectives**: Hearing loss is a prevalent sensory impairment in older adults, linked to reduced quality of life, cognitive decline, and social isolation. While it usually begins in the high-frequency range, some individuals present with isolated low-frequency hearing loss (LFHL). The long-term prognostic implications of such frequency-specific patterns remain unclear. This study aimed to evaluate the risk of long-term hearing deterioration by initial hearing loss type: LFHL, high-frequency hearing loss (HFHL), and combined-frequency hearing loss (CFHL). **Methods**: We retrospectively analyzed pure-tone audiometry (PTA) data from 10,261 patients who underwent at least two pure-tone audiometry assessments between 2011 and 2022 at a tertiary hospital. Each ear was treated as an independent observation. Hearing loss was defined as a threshold > 20 dB HL at 250, 500, 4000, or 8000 Hz. Participants were classified into normal hearing (NH), LFHL, HFHL, and CFHL groups. The outcome was a final four-frequency pure-tone average (4PTA) ≥ 40 dB HL. Logistic regression adjusted for age and sex was used, with subgroup analyses by follow-up duration. **Results**: HFHL (OR = 1.66, 95% CI: 1.47–1.89) and CFHL (OR = 2.23, 95% CI: 1.97–2.53) showed significantly higher risks of hearing loss compared with NH. LFHL did not show a significant increase (OR = 0.94, 95% CI: 0.76–1.16). These results were consistent across follow-up durations, with CFHL showing the most extensive deterioration. **Conclusion**: HFHL is a strong predictor of long-term auditory decline, and risk is further elevated with CFHL. In contrast, isolated LFHL was not associated with increased risk, suggesting relatively favorable outcomes. Frequency-specific classification may aid risk stratification and long-term monitoring strategies.

## 1. Introduction

Pure-tone audiometry (PTA) is widely regarded as the most standardized and reliable tool for the early diagnosis and prognostic assessment of hearing loss. By quantitatively measuring hearing thresholds across a range of frequencies, typically from 250 to 8000 Hz, it enables an objective evaluation of both the severity and the configuration of auditory impairment. In the early stages of hearing loss, abnormalities often appear only within specific frequency ranges; therefore, frequency-specific threshold assessment provides critical information for differential diagnosis and prognosis prediction Ref. [1]. Pure-tone audiometry is not only performed after the onset of symptoms but is also recommended as a regular screening tool for high-risk populations, including older adults, individuals with significant noise exposure, and those with a history of otologic disorders [2]. Such early screening plays a crucial role in detecting the initial patterns of hearing loss, enabling the timely identification of progressive impairment or reversible conditions, and thereby advancing the optimal timing for therapeutic intervention [3]. 

The examination is conducted in a soundproof booth using standardized equipment to measure the minimum audible intensity (threshold) for each frequency. The examiner fits the participant with headphones and presents pure tones at various frequencies, gradually increasing the sound intensity until the participant signals that the tone is perceived. The results are then used to plot a frequency-specific audiogram, which enables precise diagnosis of the type and degree of hearing loss, including low-frequency, high-frequency, and combined patterns.

Thus, pure-tone audiometry plays a pivotal role throughout the entire continuum of hearing care, including early detection of hearing loss, differential diagnosis, prognostic assessment, and evaluation of treatment outcomes. Importantly, even in the absence of prominent symptoms in the early stages, hearing loss can already affect multiple domains such as speech perception, social interaction, and cognitive function, underscoring the importance of early screening. By quantitatively measuring thresholds across a wide range of frequencies, pure-tone audiometry can detect even subtle abnormalities, making it highly effective for the early identification of pathological changes [4]. 

The pattern of frequency-specific hearing loss provides important clues for identifying the underlying etiology. Low-frequency hearing loss is most often associated with conductive disorders, such as Eustachian tube dysfunction or acute otitis media, which can generally be resolved with medical or surgical treatment if detected early [5]. However, there are also fluctuating forms, such as Ménière’s disease, and permanent types, such as sudden sensorineural hearing loss, that may lead to irreversible impairment [6]. Low-frequency hearing loss can result from a variety of etiologies, including conductive disorders (e.g., Eustachian tube dysfunction, otitis media with effusion, cholesteatoma), sensorineural conditions (e.g., Ménière’s disease, sudden hearing loss, genetic syndromes), autoimmune or inflammatory diseases, viral infections, and less commonly, vascular or neoplastic causes [7,8,9]. Identifying the underlying pathology is therefore essential for determining prognosis and guiding appropriate treatment strategies. In contrast, high-frequency loss or combined-type impairment is more likely to progress to long-term deterioration, necessitating tailored follow-up and preventive strategies. Early identification in such cases can help prevent secondary consequences of hearing loss, including communication difficulties, social isolation, and cognitive decline, thereby contributing to the preservation of quality of life. Furthermore, regular monitoring from the stage of mild hearing loss allows for a more accurate determination of the timing and necessity of rehabilitative interventions, such as hearing aid fitting [10]. 

Age-related hearing loss typically begins in the high-frequency range and gradually extends to the mid- and low-frequency ranges. This pattern is understood to occur because the basal turn of the cochlea, which is responsible for processing high-frequency sounds, is particularly vulnerable to metabolic and mechanical damage [11,12]. However, the initial frequency of onset and the progression pattern of hearing loss can vary among individuals. In some patients, low-frequency loss occurs first, which may undergo spontaneous recovery, although in certain cases it subsequently progresses to involve the high-frequency range over time [13,14,15]. In particular, acute low-frequency hearing loss may be reversible; however, in some cases, it can recur or progress to high-frequency loss, warranting careful monitoring [16,17]. In addition, combined hearing loss involving both low- and high-frequency impairment has been reported to have a poorer prognosis than isolated loss [14,18]. 

Nevertheless, most previous studies have focused on high-frequency hearing loss (HFHL) or short-term recovery, with limited systematic analyses of the long-term prognosis of low-frequency hearing loss (LFHL). In clinical practice, LFHL is often regarded as a transient abnormality; however, in some patients, it recurs or progresses to high-frequency loss, and it has been suggested that it may serve as a prodromal sign in high-risk populations. Furthermore, although combined-frequency hearing loss (CFHL), in which LFHL and HFHL coexist, has been reported to be associated with a poorer prognosis, large-scale quantitative analyses on this condition remain scarce.

Therefore, this study aimed to empirically investigate how the risk of long-term hearing deterioration varies according to the type of initial frequency-specific hearing abnormality, based on real-world clinical data. Specifically, we compared the prognoses of patients with LFHL, HFHL, and CFHL to quantitatively assess the impact of the initial abnormality pattern on subsequent auditory function decline. This approach aims to clarify the prognosis of isolated LFHL and confirm the increased risk associated with CFHL, thereby providing evidence for the clinical utility of frequency-based classification systems and informing early intervention strategies.

## 2. Materials and Methods

### 2.1. Participants

This retrospective analysis was conducted using pure-tone audiometry data from the Hearing Big Data Center (HBDC), which integrates data from five tertiary hospitals in South Korea. For this study, only data derived from outpatient visits to the Department of Otorhinolaryngology at Wonju Severance Christian Hospital, Yonsei University, between 2011 and 2022, were included. The study was approved by the Institutional Review Board of Yonsei University Wonju College of Medicine (IRB No. CR325319), and the requirement for informed consent was waived due to the retrospective nature of the data analysis.

### 2.2. Procedures

From a total dataset comprising 27,912 audiometric records, we included data from 10,261 individuals who had undergone pure-tone audiometry on at least two separate occasions. Each ear was treated as an independent observation, thereby increasing the total number of observations and allowing quantitative analyses to be performed separately for the right and left ears.

The initial hearing status was determined using the four-frequency pure-tone average (4PTA), calculated from thresholds at 0.5, 1, 2, and 4 kHz. Participants with a 4PTA of less than 40 dB HL were selected, resulting in 12,249 baseline-eligible ears. Of these, 10,675 ears had sufficient follow-up and were included in the final analytic cohort.

In this study, hearing impairment was defined according to international standards proposed by the World Health Organization (WHO) and the American Academy of Otolaryngology–Head and Neck Surgery (AAO-HNS) as a pure-tone threshold exceeding 20 dB HL [19,20]. Accordingly, group classification was based strictly on individual thresholds at 250 Hz, 500 Hz, 4000 Hz, and 8000 Hz, using the 20 dB cutoff. Specifically, ears with thresholds <20 dB HL at all four frequencies were categorized as normal hearing (NH). Ears with thresholds ≥ 20 dB HL at both 250 and 500 Hz and <20 dB HL at both 4000 and 8000 Hz were defined as low-frequency hearing loss (LFHL). Ears with thresholds ≥ 20 dB HL at both 4000 and 8000 Hz and <20 dB HL at both 250 and 500 Hz were defined as high-frequency hearing loss (HFHL). Ears with thresholds ≥ 20 dB HL at 250, 500, 4000, and 8000 Hz simultaneously were defined as combined-frequency hearing loss (CFHL). This classification system was designed to characterize the initial hearing abnormality pattern according to the affected frequency range.

The follow-up period was defined as the interval between the first and last audiometric examinations and was stratified into three categories: <1 year, 1–4 years, and 5–10 years (hereafter denoted as “≥5 years” in figures and tables). Final hearing status was determined using the 4PTA criterion: <40 dB classified as normal hearing and ≥40 dB as hearing loss. Thus, the 20 dB threshold was used exclusively for group classification, whereas the 40 dB cutoff was applied to define baseline eligibility and final hearing status.

**Figure 1:**
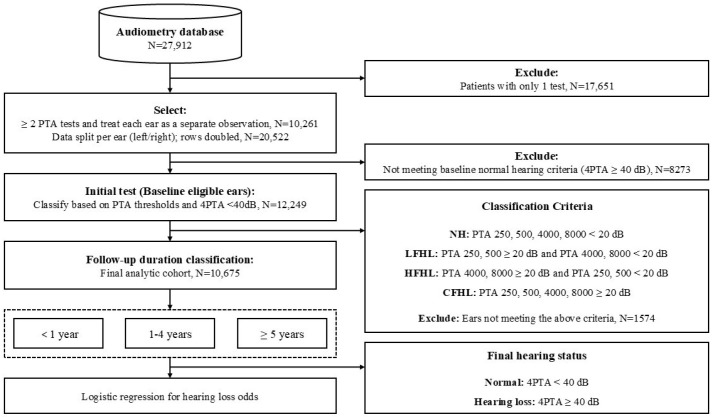
Flowchart of the study procedure for participant selection, classification, and outcome analysis. Participants with only one pure-tone audiometry test were excluded. Ears not meeting baseline normal hearing criteria (4PTA < 40 dB HL at initial test) or not meeting any of the classification criteria were also excluded. Participants were classified into four hearing groups based on their initial pure-tone audiometry thresholds: normal hearing (NH), low-frequency hearing loss (LFHL), high-frequency hearing loss (HFHL), and combined-frequency hearing loss (CFHL). Classification criteria were defined as thresholds ≥ 20 dB HL at 250/500 Hz (LFHL), 4000/8000 Hz (HFHL), or both (CFHL). The NH group included participants with thresholds < 20 dB HL at all four frequencies (250, 500, 4000, and 8000 Hz).

Logistic regression analysis was performed to assess the relative risk of hearing deterioration according to the initial hearing abnormality, with NH as the reference group (Figure 1). Bone conduction thresholds were not evaluated in this study [21]. 

Two supplementary analyses were conducted. First, to evaluate whether the degree of baseline hearing loss influenced subsequent deterioration, annual regression slopes of 4PTA progression within 10 years were calculated, stratified by baseline hearing patterns (NH, LFHL, HFHL, and CFHL). Second, to address potential bias related to follow-up, the distribution of baseline hearing patterns was examined among patients who did not undergo a second audiometric examination within 2 years.

## 3. Statistical Analysis

### 3.1. Group Comparisons

Demographic characteristics and hearing thresholds were compared among groups classified according to the type of initial hearing abnormality. The normality of continuous variables was evaluated using the Shapiro–Wilk test, and homogeneity of variances was assessed using Levene’s test. Both assumptions were formally violated for all audiometric thresholds (all *p* < 0.001). Therefore, nonparametric tests (Kruskal–Wallis with Holm-adjusted post hoc comparisons) were employed for statistical inference. However, to facilitate interpretability and comparability with prior studies, continuous variables are presented as mean ± standard deviation (SD), while categorical variables are reported as frequency and percentage (*n*, %).

### 3.2. Logistic Regression for Hearing Loss Prediction

Logistic regression was employed to predict the risk of hearing loss. The type of initial hearing status (NH, LFHL, HFHL, CFHL) was used as the independent variable, and hearing loss status—defined as a 4PTA ≥ 40 dB at the final examination—served as the dependent variable. Odds ratios (ORs) were calculated both before and after adjustment for potential confounders, including sex and age.

### 3.3. Subgroup Analyses by Follow-Up Duration

To examine differences in the effect according to follow-up duration, subgroup analyses were performed by categorizing the interval between the first and last examinations into <1 year, 1–4 years, and ≥5 years.

### 3.4. Statistical Software

All statistical analyses were performed using Python 3.12.3 (conda-forge package, 64-bit, AMD64). Data processing was conducted with pandas (v2.2.2) and numpy (v1.26.4), statistical analyses were performed using scipy (v1.13.0) and statsmodels (v0.14.4), and data visualization was carried out with matplotlib (v3.9.0) and seaborn (v0.13.2).

## 4. Results

Comparison of demographic characteristics among the four groups classified by initial hearing type revealed that the CFHL group had the highest mean age (62.6 years), while the NH group had the lowest (57.2 years). Regarding sex distribution, the proportion of males was highest in the HFHL group (65.2%), whereas the NH and LFHL groups had relatively higher proportions of females (44.5% and 48.2%, respectively) (Table 1).

Examination of the initial hearing thresholds showed that the LFHL group exhibited the greatest impairment at low frequencies (250 Hz and 500 Hz), whereas the HFHL group demonstrated pronounced elevation of thresholds in the high-frequency range above 3000 Hz. The CFHL group displayed elevated thresholds across both low and high frequencies, indicating a broad pattern of hearing loss.

At the final hearing assessment, each group either maintained a configuration of hearing loss consistent with its initial classification or exhibited further deterioration. Notably, the CFHL group had the highest final thresholds across all frequencies, suggesting a generalized decline in hearing sensitivity over time. Importantly, deterioration at low frequencies (250 and 500 Hz) during follow-up was observed only in the HFHL group, while the CFHL group, despite elevated thresholds at baseline, did not show further decline in this range. Differences among the groups were statistically significant for all variables (*p* < 0.001).

In the univariable logistic regression analysis, both the HFHL and CFHL groups showed a significantly higher risk of hearing loss compared with the NH group (Table 2). The CFHL group exhibited the highest odds ratio (OR) at 3.37 (95% CI: 2.87–3.95, *p* < 0.001), followed by the HFHL group with an OR of 2.04 (95% CI: 1.72–2.41, *p* < 0.001). In contrast, the LFHL group had an OR of 0.90 (95% CI: 0.66–1.22, *p* = 0.48), showing no statistically significant difference from the NH group.

These trends persisted in the multivariable analysis after adjusting for age and sex. The CFHL group retained the highest risk, with an adjusted OR of 3.00 (95% CI: 2.60–3.46, *p* < 0.001), followed by the HFHL group with an adjusted OR of 1.83 (95% CI: 1.59–2.11, *p* < 0.001). The LFHL group again showed no significant association with hearing loss after adjustment (adjusted OR 0.85, 95% CI: 0.75–1.14, *p* = 0.47). In the multivariable model, age was significantly associated with an increased risk of hearing loss (*p* < 0.01), whereas sex was not a significant factor in any group.

**Figure 2:**
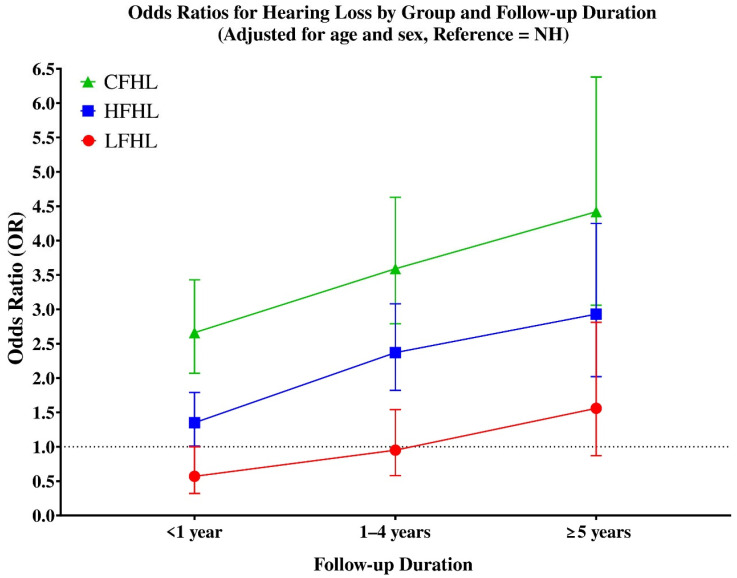
Adjusted odds ratios (ORs) for hearing loss by initial hearing group across follow-up durations (<1 year, 1–4 years, and ≥5 years). Groups are defined as follows: LFHL, low-frequency hearing loss (red circles); HFHL, high-frequency hearing loss (blue squares); and CFHL, combined-frequency hearing loss (green triangles). Error bars indicate 95% confidence intervals. All ORs were estimated using logistic regression adjusted for age and sex. The dotted horizontal line at OR = 1 represents the reference value compared with the normal hearing (NH) group.

Line graph showing adjusted odds ratios (ORs) for hearing loss by initial hearing group across different follow-up durations (<1 year, 1–4 years, and ≥5 years) (Figure 2).

Analysis of hearing loss risk according to follow-up duration (Table 3) showed distinct temporal patterns across groups. In the LFHL group, the risk was not significantly elevated within the first year (OR = 0.57, 95% CI: 0.58–1.25), but became significant after longer follow-up, with increased odds at both 1–4 years (OR ≈ 1.95, 95% CI: 1.21–1.88, *p* < 0.05) and ≥5 years (OR ≈ 1.56, 95% CI: 1.75–2.69, *p* < 0.05).

The HFHL group demonstrated a non-significant trend during the first year (OR = 1.35, 95% CI: 0.67–1.31), but showed markedly elevated risks thereafter, with ORs of 2.37 (95% CI: 1.48–2.18, *p* < 0.05) at 1–4 years and 2.93 (95% CI: 1.99–2.91, *p* < 0.05) at ≥5 years.

The CFHL group exhibited the highest long-term risk overall. While the increase was not statistically significant in the first year (OR = 2.66, 95% CI: 0.71–1.58), the odds rose sharply thereafter, reaching 3.59 (95% CI: 1.31–2.17, *p* < 0.05) at 1–4 years and 4.42 (95% CI: 1.62–2.70, *p* < 0.05) at ≥5 years. These results highlight that CFHL carries the most severe long-term prognosis, while HFHL also represents a strong predictor of progressive hearing loss, and LFHL—although relatively stable in the short term—still poses an increased risk over longer follow-up duration.

**Figure 3:**
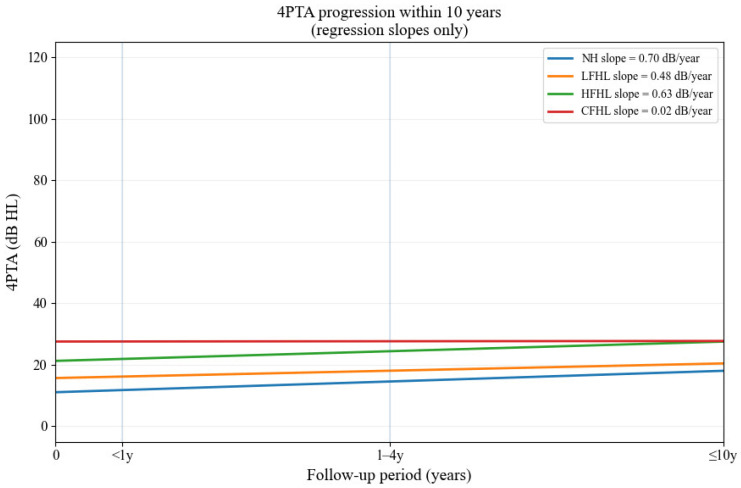
Regression slopes of 4PTA progression within 10 years across baseline hearing patterns. Regression slopes of 4PTA progression within 10 years according to baseline hearing patterns. NH showed the fastest decline, while CFHL demonstrated minimal additional deterioration.

When evaluating the relationship between baseline hearing status and subsequent deterioration, regression slopes of 4PTA revealed distinct progression patterns. NH exhibited the fastest annual decline (0.70 dB/year), while HFHL and LFHL showed intermediate progression (0.63 and 0.48 dB/year, respectively). CFHL demonstrated a markedly attenuated slope (0.02 dB/year), suggesting that the limited progression may be attributable to a ceiling effect, as these patients already presented with elevated thresholds at baseline. This analysis supports the reviewer’s concern that the extent of existing hearing loss influences subsequent deterioration and provides additional context to the odds ratio findings (Figure 3).

**Figure 4:**
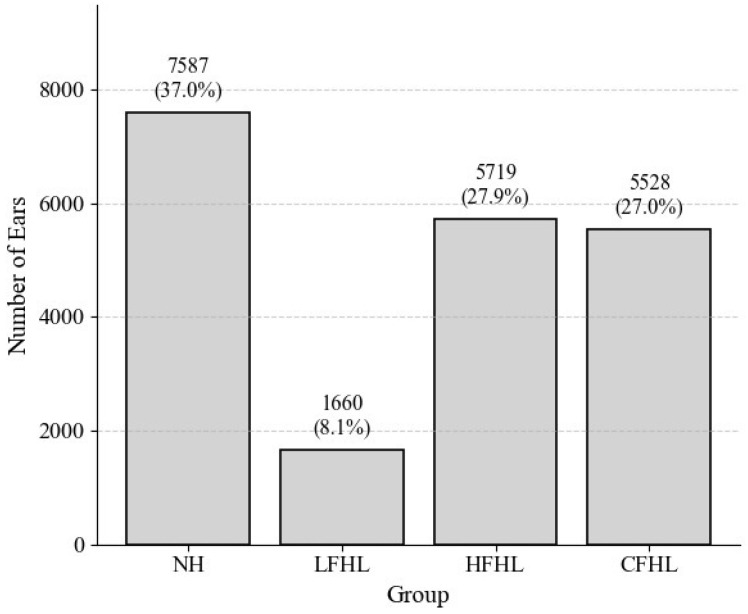
Distribution of hearing patterns among patients without a follow-up test within 2 years. Distribution of NH, LFHL, HFHL, and CFHL in patients who did not undergo a second hearing test within 2 years. LFHL accounted for only 8.1%, whereas HFHL and CFHL comprised larger proportions.

To address the reviewer’s concern regarding potential bias from patients who did not undergo a second hearing test within 2 years, we examined the distribution of hearing patterns in this subgroup (Figure 3). Among these patients, NH constituted the largest proportion (37.0%), followed by HFHL (27.9%) and CFHL (27.0%). LFHL accounted for only 8.1%, suggesting that temporary conditions associated with LFHL may have led to more frequent referrals for follow-up testing, thereby reducing its relative representation in the non-follow-up group (Figure 4).

## 5. Discussion

This study quantitatively analyzed the impact of different frequency-specific hearing loss patterns, identified through initial pure-tone audiometry, on the long-term risk of hearing deterioration using a large-scale retrospective dataset. The analysis demonstrated that high-frequency hearing loss (HFHL) is a significant predictor of future hearing loss, while combined-frequency hearing loss (CFHL), involving both low- and high-frequency impairment, was associated with the most severe hearing loss across the entire frequency spectrum and the poorest long-term prognosis. In contrast, isolated low-frequency hearing loss (LFHL) was not significantly associated with long-term hearing deterioration and tended to present a relatively favorable prognosis.

By adopting a dichotomized classification system that designated 250/500 Hz as representative low frequencies and 4000/8000 Hz as representative high frequencies, we established clinically clear and interpretable diagnostic criteria. This approach enabled the sensitive detection of early changes in hearing patterns and allowed for a quantitative evaluation of their impact on long-term hearing outcomes, thereby providing evidence that may be applicable in real-world clinical practice.

The long-term patterns of hearing change varied markedly according to the type of initial hearing abnormality. In cases presenting with LFHL alone, the overall prognosis tended to be favorable; however, some patients experienced progression of hearing loss to the high-frequency range or across the entire frequency spectrum over time, or demonstrated recurrent episodes, highlighting the need for long-term monitoring. LFHL is often associated with reversible conditions such as Ménière’s disease or Eustachian tube dysfunction, with many cases showing recovery. Nevertheless, long-term progression to high-frequency loss or recurrent deterioration has also been reported [6,14]. Accordingly, while isolated LFHL may have a relatively favorable prognosis, the possibility of progression cannot be excluded, and continued follow-up is recommended.

In this study, we found distinct long-term prognostic differences according to the type of initial hearing abnormality. High-frequency hearing loss (HFHL) was associated with gradual progression involving the mid- and low-frequency ranges, while combined-frequency hearing loss (CFHL) exhibited the most rapid and severe deterioration across the entire spectrum, identifying it as the group at highest risk for long-term decline. In contrast, low-frequency hearing loss (LFHL) generally showed a more favorable prognosis, often stabilizing or recovering over time. Nevertheless, even among the normal-hearing (NH) group, a subset of patients developed new-onset hearing loss during follow-up, though the incidence was lower compared with the abnormal hearing groups. These findings underscore the prognostic value of initial hearing patterns and support the need for tailored monitoring and intervention strategies.

The poor prognosis observed in the HFHL group can be explained by the anatomical and physiological vulnerability of the cochlear basal turn, which processes high-frequency sounds but has relatively limited blood supply and structural resilience. This region is therefore more susceptible to age-related degeneration (presbycusis), chronic noise exposure, ototoxic medications (e.g., aminoglycosides, diuretics), and metabolic disorders such as diabetes mellitus and hypertension. Once damaged, the regenerative capacity of cochlear hair cells is extremely limited, consistent with previous studies that identify HFHL as an early marker of irreversible auditory decline and a major predictor of progressive hearing loss [22,23,24]. 

This finding suggests that exclusively high-frequency hearing loss is not only a strong predictor of further deterioration at high frequencies but may also serve as an early marker of subsequent low-frequency involvement. Such a progression pattern supports the view that HFHL can evolve into a pantonal configuration over time, underscoring the need for long-term monitoring even in patients who initially present with isolated HFHL.

In contrast, LFHL often arises from reversible conditions such as Ménière’s disease, Eustachian tube dysfunction, or acute infections, which explains its relatively favorable long-term outcomes. These conditions may allow recovery through appropriate treatment or spontaneous resolution. Low-frequency or flat hearing loss has also been associated with acoustic trauma, as reported by Littlefield and Brungart [25], suggesting that LFHL may arise from both reversible and trauma-related mechanisms. Prior research has reported that LFHL can recur or progress to HFHL, indicating that it should not be regarded merely as a transient abnormality but rather as a potential precursor to progressive long-term hearing loss [26,27]. Our findings are consistent with these reports, reinforcing the need for continuous and systematic follow-up in patients with isolated LFHL.

Demographic analysis further revealed that increasing age and male predominance were associated with greater severity of hearing abnormality. These trends align with prior studies linking HFHL and CFHL to age, occupational noise exposure, smoking, cardiovascular disease, and other comorbidities, which are more prevalent among older men [28,29,30]. This suggests that risk prediction models for hearing loss should integrate not only audiometric thresholds but also demographic, medical, and lifestyle factors to improve accuracy and clinical utility.

Among all groups, CFHL demonstrated the poorest prognosis, suggesting that this pattern reflects a generalized decline in cochlear function, possibly driven by overlapping pathophysiological mechanisms. In clinical practice, CFHL is often considered a ‘mixed abnormality’ or pantonal hearing loss, a configuration that may be overlooked yet carries significant risk. Patients in this group may therefore require more proactive management, including early hearing aid fitting, participation in pharmacological trials for hearing preservation, and individualized timing of auditory rehabilitation.

Furthermore, CFHL represents a pantonal pattern of hearing loss involving both low- and high-frequency ranges, and in clinical practice it is often referred to as a ‘mixed abnormality’. This configuration is easily overlooked and may pose greater diagnostic challenges compared with isolated low- or high-frequency impairments. Accordingly, interpretation of pure-tone audiometry results should not rely solely on the conventional four-frequency average (4PTA) but should also incorporate frequency-specific threshold profiling and recognition of atypical patterns. Such a multifaceted approach may facilitate earlier detection and intervention in CFHL, ultimately playing a critical role in preventing long-term deterioration in patients’ quality of life.

Among all groups, CFHL demonstrated the poorest prognosis and may represent not simply a frequency-specific abnormality but a generalized decline in cochlear function. Patients with this pattern may therefore require more proactive management, including early hearing aid fitting, individualized rehabilitation planning, and consideration for participation in hearing-preservation trials. These findings emphasize that initial hearing-loss patterns are important clinical indicators that warrant tailored monitoring and intervention strategies.

This stratification provides a practical tool for audiologists and otolaryngologists to prioritize follow-up intensity and counseling strategies according to the patient’s initial audiometric configuration. Integrating audiometric profile-based risk prediction into routine clinical workflows may support shared decision-making, personalized patient education, and timely rehabilitation planning. Although all patients benefit from regular monitoring, those with CFHL patterns may warrant earlier audiological rehabilitation and medical intervention, whereas isolated LFHL may be monitored more conservatively without immediate intervention.

Beyond audiometric patterns alone, it is likely that demographic and clinical factors such as age, sex, cardiovascular or metabolic comorbidities, and history of noise exposure further influence the risk of long-term hearing deterioration. Prior studies have demonstrated associations between hearing decline and systemic conditions including diabetes, hypertension, and smoking [28,29,30]. While the present analysis focused on frequency-specific profiles, integrating these risk factors into future predictive models may enhance clinical utility and allow for more individualized patient counseling and management.

In addition, our supplementary analyses provided further insight into the dynamics of long-term hearing change. The evaluation of annual regression slopes demonstrated that the rate of deterioration varied according to the baseline hearing pattern, with NH showing the fastest decline, whereas CFHL exhibited minimal additional deterioration, likely due to a ceiling effect from already elevated thresholds. This highlights that not only the presence but also the magnitude of baseline hearing loss influences subsequent progression. Furthermore, when examining patients who did not undergo a second hearing test within 2 years, LFHL was underrepresented compared with HFHL and CFHL. This finding suggests that LFHL cases may have been more frequently referred for follow-up due to their potentially reversible nature, which may partially explain the relatively favorable long-term prognosis observed in this group.

## 6. Limitation

This study has inherent limitations in causal interpretation due to its retrospective design based on single-center data. In addition, key confounding variables—such as history of noise exposure, occupational risk factors, underlying otologic diseases, and hearing aid use—were not available and thus could not be statistically adjusted for. Furthermore, because the study focused on objective changes in hearing thresholds, it did not include information on clinical symptoms (e.g., tinnitus, dizziness), subjective auditory sensitivity, or the actual degree of functional impairment in daily life. As a result, the analysis linking hearing deterioration to quality of life was limited.

Another limitation is that patients with low-frequency hearing loss were not stratified by underlying etiology (conductive vs. sensorineural). Conductive LFHL can be reversible with medical or surgical treatment, whereas sensorineural LFHL may represent irreversible or progressive pathology. The absence of this distinction may have introduced bias and restricts the generalizability of our findings. Future research, particularly through multi-center prospective studies, is needed to more precisely identify risk factors and clarify long-term outcomes.

Finally, the study did not stratify patients by demographic or systemic risk factors (e.g., age, sex, diabetes, cardiovascular disease), which are known to influence hearing outcomes. Although we discussed these associations conceptually, their absence from the statistical model limits the scope of our conclusions. Future multi-center, prospective studies incorporating these risk factors are needed to strengthen predictive validity and clinical applicability.

## 7. Conclusions

Based on more than 10,000 real-world clinical records, this study quantified the long-term risk of hearing loss according to the type of initial hearing abnormality and empirically demonstrated that frequency-specific classification can serve as a clinically useful prognostic criterion beyond simple diagnosis. By subdividing hearing loss into frequency-specific patterns rather than simplifying it into a single average threshold, our approach provides richer and more precise clinical information for predicting progression compared with the conventional four-frequency average (PTA). Such classification offers a practical foundation for developing personalized strategies, including hearing aid prescription, auditory rehabilitation, pharmacological treatment, and vocational counseling.

HFHL was identified as a significant predictor of progressive hearing decline, while CFHL showed the poorest prognosis, underscoring the need for proactive intervention in this group. In contrast, LFHL generally exhibited a more favorable prognosis, although recurrent or progressive cases were observed, highlighting the necessity of systematic long-term follow-up.

These findings also provide a scientific basis for strengthening screening programs for high-risk groups (e.g., older men), establishing structured follow-up systems for isolated LFHL, and developing tailored management strategies for CFHL. Ultimately, frequency-based hearing classification systems have the potential to evolve from tools for early diagnosis into robust clinical instruments for preventing hearing loss and mitigating its broader consequences, including cognitive decline, social isolation, and reduced quality of life. Future studies employing multi-center prospective cohorts are warranted to incorporate multifaceted analyses, including the underlying pathophysiological mechanisms, associations with accompanying symptoms such as tinnitus and dizziness, treatment responsiveness, and the effects of hearing aid use.

## Figures and Tables

**Table 1:** Demographic characteristics and hearing thresholds at initial and final assessments across frequency-based hearing groups.

Variable	NH (*n* = 4054)	LFHL (*n* = 915)	HFHL (*n* = 2957)	CFHL (*n* = 2749)
Age (Mean ± SD)	57.2 ± 15.8	58.8 ± 16.9	62.5 ± 14.2	62.6 ± 16.7
Sex (n, %)				
Men	2250 (55.5%)	474 (51.8%)	1929 (65.2%)	1614 (58.7%)
Women	1804 (44.5%)	441 (48.2%)	1028 (34.8%)	1135 (41.3%)
Initial hearing level (dB)				
PTA_AC_250	7.98 ± 4.97	25.28 ± 7.70	10.04 ± 4.46	27.57 ± 8.91
PTA_AC_500	7.73 ± 4.90	24.92 ± 7.16	9.97 ± 4.48	26.73 ± 7.70
PTA_AC_1000	7.33 ± 5.78	13.14 ± 8.74	12.61 ± 7.71	23.11 ± 9.98
PTA_AC_2000	7.00 ± 6.07	11.10 ± 7.29	15.86 ± 10.53	22.75 ± 9.72
PTA_AC_3000	6.85 ± 5.30	9.13 ± 5.27	29.16 ± 15.49	29.79 ± 12.93
PTA_AC_4000	7.44 ± 5.30	9.42 ± 4.99	37.19 ± 16.62	35.78 ± 15.17
PTA_AC_8000	7.50 ± 5.32	9.40 ± 5.02	41.17 ± 18.79	43.14 ± 20.11
Final hearing level (dB)				
PTA_AC_250	14.11 ± 14.23	19.70 ± 14.35	17.33 ± 16.57	24.87 ± 17.98
PTA_AC_500	14.02 ± 14.55	19.71 ± 14.60	17.49 ± 17.48	25.08 ± 18.76
PTA_AC_1000	12.84 ± 15.18	14.92 ± 15.10	18.35 ± 18.45	24.48 ± 20.30
PTA_AC_2000	13.25 ± 16.11	14.79 ± 15.26	20.69 ± 19.28	24.44 ± 20.46
PTA_AC_3000	15.05 ± 17.90	15.92 ± 16.64	31.23 ± 22.22	30.85 ± 22.68
PTA_AC_4000	17.19 ± 20.09	18.43 ± 19.31	37.18 ± 23.73	36.19 ± 24.55
PTA_AC_8000	18.53 ± 22.00	19.11 ± 20.24	41.31 ± 26.02	42.23 ± 27.94

NH = normal hearing; LFHL = low-frequency hearing loss; HFHL = high-frequency hearing loss; CFHL = combined-frequency hearing loss; SD = standard deviation; n = number.

**Table 2:** Univariable and Multivariable Logistic Regression Results for Predicting Hearing Loss Risk by Hearing Loss Group (Reference: Normal Hearing).

Group	Univariable	Multivariable		
	OR (95% CI)	Adjusted OR (95% CI)	Age OR (95% CI)	Sex OR (95% CI)
NH (Reference)	1.00	1.00	-	-
LFHL	0.90 (0.66–1.22)	0.85 (0.75–1.14)	1.03 (1.03–1.04) *	0.82 (0.65–1.05)
HFHL	2.04 (1.72–2.41) *	1.83 (1.40–1.81) *	1.03 (1.02–1.03) *	1.09 (0.92–1.30)
CFHL	3.37 (2.87–3.95) *	3.00 (1.86–2.40) *	1.02 (1.02–1.03) *	0.95 (0.81–1.11)

* *p*-value < 0.05, considered statistically significant. OR = Odds Ratio; CI = Confidence Interval; HL = Hearing Loss; NH = Normal Hearing; LFHL = Low-Frequency Hearing Loss; HFHL = High-Frequency Hearing Loss; CFHL = Combined-Frequency Hearing Loss. Reference group = NH. Univariable models: unadjusted logistic regression for hearing loss risk by hearing group. Multivariable models: adjusted for age and sex. “Age OR” and “Sex OR” represent the odds ratios for hearing loss per 1-year increase in age and for male sex (vs female), respectively. OR > 1 indicates increased odds of hearing loss; OR < 1 indicates decreased odds.

**Table 3:** Odds ratios for hearing loss by hearing group and follow-up duration (reference: NH).

Graph 1.	<1 Year OR (95% CI)	1–4 Years OR (95% CI)	≥5 Years OR (95% CI)
NH (Reference)	1.00	1.00	1.00
LFHL	0.57 (0.32–1.00)	0.95 (0.58–1.54) *	1.56 (0.87–2.81) *
HFHL	1.35 (1.01–1.79)	2.37 (1.82–3.08) *	2.93 (2.02–4.25) *
CFHL	2.66 (2.07–3.43)	3.59 (2.79–4.63) *	4.42 (3.062–6.38) *

* *p*-value < 0.05, considered statistically significant. OR = Odds Ratio; CI = Confidence Interval; HL = Hearing Loss; NH = Normal Hearing; LFHL = Low-Frequency Hearing Loss; HFHL = High-Frequency Hearing Loss; CFHL = Combined-Frequency Hearing Loss. ORs were calculated using logistic regression models with NH as the reference group within each follow-up duration category. Values are presented as OR (95% CI). OR > 1 indicates increased odds of hearing loss compared with NH; OR < 1 indicates decreased odds. Follow-up duration categories were defined as <1 year, 1–4 years, and ≥5 years from the baseline audiometric assessment.

## Data Availability

The audiometric dataset used in this study was obtained from the Hearing Big Data Center (HBDC), which collects data from five tertiary hospitals in South Korea. Among these, only the dataset from Wonju Severance Christian Hospital, Yonsei University, was used for the present analysis. Data are not publicly available due to patient privacy protection and institutional review board (IRB) restrictions, but may be available from the corresponding author upon reasonable request and with permission from the institution.

## Abbreviations

The following abbreviations are used in this manuscript:

PTA	Pure-Tone Audiometry
4PTA	Four-Frequency Pure-Tone Average
LFHL	Low-Frequency Hearing Loss
HFHL	High-Frequency Hearing Loss
CFHL	Combined-Frequency Hearing Loss
NH	Normal Hearing
